# The influence of expanded carrier screening in assisted reproductive techniques: changed the ‘game’– review

**DOI:** 10.1097/MS9.0000000000000657

**Published:** 2023-04-17

**Authors:** Petros Drettas, Vasileios Tatanis, Chara Spiliopoulou, Georgios Adonakis, Evangelos Liatsikos

**Affiliations:** a Department of Urology, University of Patras, Patras, Greece; b Department of Forensic Medicine and Toxicology, School of Medicine, National and Kapodistrian University of Athens, Greece; c Department of Obstetrics and Gynecology, University Hospital of Patras, Patras, Greece

**Keywords:** assisted reproductive technique, expanded carrier screening, gamete donation, in-vitro fertilization

## Abstract

Expanded carrier screening constitutes a new scientific tool able to detect conditions that can be treated immediately after birth or during pregnancy. Its implementation could affect both the prenatal period and assisted reproductive techniques. It is strongly beneficial as it provides much useful information to future parents concerning the medical status of their offspring. In addition, the definition of ‘serious/severe’, regulating preimplantation diagnosis, donor insemination, and even the definitions of prerequisites for abortion diseases, should be reformed including all clinically severe diseases. On the other hand, controversies may arise especially regarding gamete donation. Future parents and offspring maybe informed regarding donors’ demographic and medical characteristics. This study aims to investigate the effects of the implementation of expanded carrier screening in the reformation of the definition of ‘severe/serious’ disease, the decision-making of future parents, gamete donation, and the possible new moral dilemmas that may arise.

## Introduction

Highlights

- Expanding carrier screening may lead to increased preimplantation genetic diagnosis.
- Expanding carrier screening may give information about the potential medical risks.
- The definition of ‘serious/severe’ disease should be reformed.
- Expanding carrier screening minimizes the medical risks for both donors and offspring.

Infertility is a disease of the reproductive system defined by the failure to achieve a clinical pregnancy after at least 12 months of regular unprotected sexual intercourse^1^. Based on population surveys, the prevalence of infertility ranges from 3.5 to 16.7% in developed countries and from 6.9 to 9.3% in less-developed nations, while the estimated overall median prevalence is 9%^2^.

In the 1960s and 1970s, the study of the events happening during human oocyte fertilization was remarkable, leading to the development of the Assisted Reproductive Technique (ART). IVF constituted the solution to the blocked fallopian tube and the first ‘test-tube baby’ was born in 1978. Currently, ART is associated with 2–6% of live births in European countries, counting more than 1 600 000 live births since 2017^3^.

Recently, many research developments have been realized in the field of genomics. The most notable is the ongoing transition from traditional ‘monogenic genetics’ toward comprehensive human genome testing by integrating massively parallel sequencing approaches. Currently, it is possible to elucidate the entire human genome, regardless of the initial medical indication for which a patient (accompanied by his family members) was referred for genetic testing. The technological advances are reflected in expanded carrier screening and preimplantation genetic testing (PGT).

Our purpose is to evaluate the effect of ECS and PGT in the evolution of ART practices both in terms of decision-making and in terms of moral/bioethical dilemmas that may arise for future parents, scientific bodies, and human societies.

## Expanded carrier screening

ECS refers to genetic carrier screening beyond one’s ethnicity and family history. Using ECS, single-gene disorders, that are not included in guidelines, maybe identified in low-risk populations. ECS panels constitute a more cost-effective alternative to carrier genetic testing, as it generally uses next-generation sequencing to read through hundreds of genes at one time. The inclusion of genes in ECS panels constitutes a simple and low-cost procedure, resulting in the development of commercial ECS panels capable to screen many genetic disorders^4^. Professional societies published recommendations for the responsible implementation of ECS in clinical practice, definitively with the proper and rigorous counseling and informed consent. According to the European Society of Human Genetics, genes associated with severe childhood-onset disorders should be the primary aim of ECS^5^.

ECS may lead to increased preimplantation genetic diagnosis (PGD) and less necessity for prenatal diagnosis (PND). Therefore, the rate of elective termination of pregnancy due to severe genetic disease detection could be diminished. ECS may also detect conditions able to be treated immediately after birth or during pregnancy^6^,^7^.

## Preimplantation genetic diagnosis/preimplantation genetic testing

PGD, recently renamed PGT, constitutes genetic testing of cells retrieved by biopsy from oocytes (polar bodies) or embryos created through IVF techniques. Compared to the option of PND, PGD is considered superior, as it spares them from deciding to terminate a wanted pregnancy. Initially, carrier testing was developed to detect genetic diseases in specific high-risk populations (e.g. cystic fibrosis in European-derived populations)^1^, even within a direct-to-consumer setting^8^.

PGT offers individuals with an increased risk of transmitting genetic disorders the possibility to give birth to children free of disorder carriers. In addition, it may prevent complications arising later in the development of an embryo, thus spontaneous abortion or the decision of terminating the pregnancy could be avoidable^9^. Moreover, as the risk of severe genetic diseases can be detected during the preimplantation phase, future parents could be properly orientated regarding alternative fertility options like donation or adoption. PGT has been mainly applied to disorders caused by complete or very high penetrance mutations (e.g. Huntington’s disease, cystic fibrosis). On the contrary, based on clinical observations, future parents sometimes worry about incomplete or lower-penetrance mutations involved in serious disorders, like tumors (e.g. hereditary breast and ovarian cancer) and diabetes. Female carriers of relevant BRCA mutations have a lifetime risk of up to 80% for breast cancer and up to 60% for ovarian cancer^10^.

As knowledge about the genetic parameters of several diseases is expanded, existing carrier screening does not seem to be adequate. It is estimated that at least 1 in 100 couples of the general population is at high-risk of having a child suffering from a serious recessive disorder^11^–^13^. Considering that the mentioned risk (0,25–0,50%) is almost the same as the risk of a 37-year-old woman of having a child with Down syndrome, which is already screened and accepted both bioethically and medically, the development of PGD seems integral^14^. Prenatal education of parents regarding special needs care after the birth of their offspring is also considered a critical procedure since they can embrace different fertility options.

After the development of whole-genome haplotyping approaches, the majority of inherited disease-associated variants can be identified^15^–^17^. Therefore, the meaning of serious/severe genetic disease should be reformed, considering adult-onset phenotype disorders, disorders for which the onset period cannot be distinguished by molecular testing, and diseases characterized by heterogeneity like inherited blindness and deafness^18^. Does this right oppose, for example, the status quo of the unborn baby and even worst contains an inherent wrongness according to Kantian theory, or does it enhance the welfare and well-being of the society, and in this way is morally accepted according to the Utilitarian theory?

## Serious/severe genetic disease

The word ‘serious/severe’ is almost omnipresent in daily medical practice but its role is complicated in the law framework of the ART field as it is associated with preimplantation diagnosis, donor insemination, or even the definitions of prerequisites for abortion (e.g. United Kingdom 1967/ Italy 1978/ Greece 1986/ The Netherlands 1993/). Despite the extended use of this word, a universal definition of a severe genetic disorder is difficult to be determined. Some lawmakers support the idea, that the effect on the quality of life is enough to distinguish the serious and not serious diseases^19^.

Based on the literature, physicians selecting a preconception or prenatal carrier panel should consider characteristics negatively affecting the quality of life of children as important criteria to define a severe or serious genetic disorder. Lower life expectancy, intellectual and mobile disability, and the need for repeated surgical interventions or hospitalizations are the most significant. Regardless of definition, future parents consider the painful decision of pregnancy termination in cases of ‘severe/serious’ diseases like Down syndrome, β-thalassemia, or cystic fibrosis^12^,^20^.

Monogenic diabetes constitutes a disease causing significant medical, financial, and quality-of-life consequences for both children and their families. It is a rare type of diabetes that is difficult to be diagnosed, resulting in frequently misdiagnosed patients. Consequently, these patients often receive ineffective treatment, which can aggravate disease complications. It is caused by a single-gene defect that is usually inherited in an autosomal dominant or recessive fashion while mutations causing diabetes may also be encountered in mitochondrial DNA. The exact correlation between genotype and phenotype has not been clarified but the detection of each of the mentioned mutations increases the risk of the disease. PGD of monogenic diabetes seems to be very important as it may be strongly beneficial. Firstly, the targeted treatment, which is often superior to conventional approaches, depends on the affected gene^4^,^5^,^21^. Moreover, affected family members may be detected after testing. Therefore, patients with HNF1A or HNF4A mutations maybe managed with low-dose sulphonylurea therapy, while misdiagnosed ones may potentially stop insulin injections and follow tablet regimens^22^. Furthermore, early diagnosis of MODY positively affects healthcare economics, considering that individual medical expenditures increase 2.3 times by the presence of diabetes. Economics does not constitute the main point for any reproductive genetic screening, but it should be considered. Consequently, MODY should be considered an example of a ‘new’ serious/severe genetic disease that requires the performance of ECS and PGD^23^. But does this new potential fit in the context of bioethics? Does it follow the basic principles like benevolence, or create new dilemmas despite the normative theories that medical professionals and future parents may follow or believe?

## The donor/recipient issue

Donor conception includes gamete (sperm or oocyte) and embryo donation either created ex Novo, known as ‘double donation’, or formed by the surplus embryos already created in previous ART circles^24^. Gamete donation is rapidly increasing and constitutes the first-line fertility option after the detection of potentially harmful variants in the preconception phase^9^. In 2016, the ESHRE European IVF Monitoring Consortium reported 50  467 donor sperm-intrauterine insemination (IUI) cycles and 73  927 ART treatments using donated oocytes, resulting in 6249 and 22  497 births, respectively^25^. The reasons have not been clarified but the gradual decline in the quality of human sperm, the advanced paternal and maternal age, and legal/social parameters seem to contribute to the increase in ART. The improvement in the implementation of vitrification techniques with better oocyte survival rates and the legalization of access to medically assisted reproduction (MAR) for single women and lesbians in many countries have also an important role in the increased number of gamete donations^26^. An upward trend in people traveling abroad to have ART was noticed. This phenomenon, known as ‘Fertility tourism’, is not restricted to the European Union (EU) where the free movement of citizens is allowed, but it is also expanding outside of the European borders. Lower cost treatments ameliorated quality of treatments, shorter waiting times, and a possible attempt to circumvent legal and ethical restrictions constitute some potential reasons for this phenomenon^27^.

A lack of consensus regarding the quotas for the number of patients that can be treated with a single donor or the maximum number of children born (or families created) from a single donor was noticed. The imposition of restrictions should be based on legislation (e.g. UK, HFEA guidance), professional guidelines, and MAR centers/gamete banks’ self-regulation. In some counties, the number of offspring is regulated only by the donor, provided that it remains beneath the levels set in national guidelines (e.g. UK). Due to the lack of consensus, the quotas can vary from 5 to 10 children or families per donor^28^.

International distribution of donor gametes and ‘fertility tourism’ complicate the issue as the local legislative framework varies between regions. Some countries provide national registries (e.g. HFEA in the UK), while other countries have fewer regulations and no national registry, making the enforcement of restrictions difficult^29^. For those reasons, does ‘fertility tourism’ impose a new reality not just in medical and legal prospects, but also in the moral and ethical framework that societies use to function?

The legislation concerning the anonymity of the donor differs between countries, but personally identifiable characteristics are currently and easily available in nonregulated systems^30^.

Health examinations should be performed on donors in order for their fertility to be ensured and to avoid transmission of hereditary diseases^31^. Various scientific and governmental bodies have developed different guidelines usually including medical and family history, testing for sexually transmitted diseases, and genetic screening for specific conditions (e.g. karyotyping or carrier status)^32^. Currently, ECS could be applied to recipients without distinction for parents who undergo ART without MAR and those using donor gametes^33^. Apart from medical reasons, the recipients want to be informed concerning the race, lifestyle, intelligence, education, and personality of the donor^34^. Lesbian recipients are mainly interested in characteristics facilitating normal child development and reinforcing family coherence^35^. In a survey among lesbian parents, three advantages of known donation were acknowledged by those who conceived through anonymous sperm donation: medical reasons (need for organ transplantation), the function of the donor as an extra caregiver and the possibility of communication establishment between donor and offspring^36^. On the contrary, donors are hesitant regarding the process of genetic screening^37^. Several studies suggest the information of donors regarding the possible short- and long-term emotional consequences of their donation, the emotional and physiological effects of genetic screening results, and the disclosure to family and friends. It seems that the main reason for gametes donation is also connected to that. A combination of altruism and financial interest are the motivations for gametes donors. In a recent study, only 1 in 4 donors was motivated exclusively by altruism, while the remaining donors were mainly motivated by financial compensation^38^. Since the percentage of donors motivated by pure altruism is quite low, does the term moral egoism comes into the scene, is this independent of normative forms and does the latter change the current bioethics standpoints?

Ancestry databases for genetic testing are widely utilized during the last decades^39^ even with a donor-conception section. This trend may reveal new concerns regarding the anonymity of donors. Moreover, these databases may reveal the lack of a genetic association between the social parent and the offspring, the fact that a family member has donated gametes/embryos or the identification of genetic relatives (a donor, siblings, or family) before the age set by national legislation^40^. Registries founded by donor-conceived offspring or their parents (e.g. Donor Sibling Registry, USA), or publicly funded (e.g. Donor-Conceived Register, 2004 UK) were also developed. Their aim is to facilitate the establishment of mutually desired contact between donors and donor-conceived offspring. The inability to guarantee anonymity constitutes a reality to which people must be adapted. “Fertility tourism” seems to complicate more this issue. The fact that countries allowing anonymous donation are visited by patients from countries where nonanonymous donation is applied and vice versa constitutes proof of the extent of this issue^41^,^42^. The implementation of ECS achieved a minimization of the medical risks for both donors and offspring during ART procedures. The knowledge of genetic origins could be beneficial for the assessment of genetic risks and the application of genetic therapies^28^.

## Discussion

One of the major advantages of ECS/PGD is the avoidance of repeated selective abortions, even if this requires IVF, which cannot be considered the most pleasant way to reproduce. This can satisfy one of the major objections, the ‘sanctity of human life’, in that case, the embryo’s life^43^. But apart from the generally accepted theory of the ‘relatively low moral status of the embryo’, the implementation of these techniques seems to fit with various accepted practices like the disposal or loss of spare embryos, the use of prescribed intrauterine contraceptive devices and PND. There is a strong consensus that selective abortion is morally justified when it can prevent ‘serious’ suffering for both parents and children^43^,^44^. So, under the spotlight of the ECS/PGD, should we redefine the ‘serious’/‘severe’ health issues? It is quite interesting the fact that societies already use PND and possible selective abortion, in order to avoid not only life-threatening diseases but also health issues that can compromise the quality of life of the affected person like β-thalassemia, Huntington’s disease, cystic fibrosis^9^, which perfectly fits in the field of the so-called ‘disability rights’, even if the argument is questionable^45^. There is definitively a moral and human obligation to support the rights and improve the quality of life of disabled people, in any form and in any degree, but it is a *non sequitur* to argue that this generates a claim on the part of those individuals that society must ensure that their numbers do not decrease, as Guido de Wert says. We cannot fight all contingencies of human life but since we can now recognize lower-penetrance mutations for disorders that maybe preventable and/or treatable like breast and ovarian cancer and in our days MODY diabetes, which affects millions of people globally with a huge medical and psychological cost for both patients and family members. We think that ECS/PGD should be considered a morally accepted technique, not only because it may effectively reduce morbidity and mortality in carriers^46^, but mostly because it honors and follows the basic principles of bioethics like autonomy and benevolence. From either a deontological or consequentialist point of view, the choice and the action should be considered moral and ethical in this regard. And the same principle can be applied in the case of gamete donation. It is difficult to understand the objections raised in this field especially if the recipient carries one or more potentially harmful variants. The right ‘not to know’ for the donor, for any reason, should be embraced and respected. But where we should drown the line between the right of the donor and the right of the recipient and even more important the interests of the future children, which still constitutes the fundamental value is assisted reproduction. It is also difficult to understand how the knowledge of such important medical information constitutes a contrast to the motive of the donor, especially in the upcoming era of genetic therapies. The chance of applying but not disclosing that information, obviously after rigorous information and consent maybe it is a solution. After all, in most countries, the meeting of the recipient/donor is not allowed or is granted to the donor, and in Germany it is excluded^30^. The ‘fertility tourism’ phenomenon, often in a not legal framework originated from the intrinsic need of human beings to procreate, often at any cost, constitutes a huge new challenge that will require new extensive mostly legal actions which are beyond the aim of work.

## Conclusion

Undoubtedly IVF, since the beginning had a major effect on human societies mostly because for the first time in history, life does not start inside the human body. Governments had to create new laws, and scientific bodies to adopt and embrace new ethical standards. For those reasons, it can be considered one of the main game changers in medical history, not only in terms of technology but also in terms of legal, ethical, and moral standards. The recent total decoding of the human genome is another major breakthrough in medicine because it gives the potential of understanding the real origins of life both in terms of health and disease.

Biomedical technologies are neither morally nor socially neutral but deeply influenced by medical, cultural, and political norms. Even if the major aim of this work is to review the effect of ECS/PGD on medical decisions we realized that normative discussions that usually surround different forms of ART should be included. We do understand that further research and work need to be done, especially since there is not a universally accepted moral theory but we do acknowledge that ECS/PGD looks like a game changer.

## Ethical approval

NA.

## Inform consent

NA.

## Sources of funding

This research received no specific grant from any funding agency.

## Author contribution

P.D.: project development, data collection and data analysis, manuscript drafting; V.T.: data collection and data analysis, manuscript revision; C.S.: project development, and critical revision; G.A.: project development, and critical revision; E.L.: project development, and critical revision.

## Conflicts of interest disclosure

The authors declared no conflict of interest.

## Guarantor

Petros Drettas.

## Provenance and peer review

Not commissioned, externally peer reviewed.
